# Sex-specific associations between serum lipids and hemostatic factors: the cross-sectional population-based KORA-fit study

**DOI:** 10.1186/s12944-022-01757-0

**Published:** 2022-12-21

**Authors:** Johannes Vogel von Falckenstein, Dennis Freuer, Annette Peters, Margit Heier, Daniel Teupser, Jakob Linseisen, Christa Meisinger

**Affiliations:** 1 Epidemiology, Medical Faculty, University of Augsburg, University Hospital of Augsburg, 86156 Augsburg, Germany; 2 Helmholtz Zentrum München, Institute for Epidemiology, Ingolstädter Landstr. 1, 85764 Neuherberg, Germany; 3 Institute for Medical Information Processing, Biometry and Epidemiology, Ludwig-Maximilians-Universität München, Munich, Germany; 4 German Center for Cardiovascular Disease (DZHK), Partner Site Munich Heart Alliance, Munich, Germany; 5 KORA Study Centre, University Hospital Augsburg, Augsburg, Germany; 6 Institute for Laboratory Medicine, Ludwig-Maximilians-Universität München, Munich, Germany

**Keywords:** Serum lipids, Blood coagulation, KORA study, General population, Sex differences

## Abstract

**Background:**

Studies on the associations between lipid parameters and different hemostatic factors in men and women from the general population are scarce. It was therefore examined whether there are possible relationships between routinely measured serum lipids (total cholesterol, HDL-cholesterol, non-HDL-cholesterol, LDL-cholesterol, and triglycerides) and different hemostatic factors (activated partial thromboplastin time (aPTT), fibrinogen, factor VIII, antithrombin III (AT III), protein C, protein S, and D-dimer).

**Methods:**

The analysis was based on data from the Cooperative Health Research in the Region of Augsburg (KORA)-Fit study, which included 805 participants (378 men, 427 women) with a mean age of 63.1 years. Sex-specific associations between serum lipids and coagulation factors were investigated using multivariable linear regression models.

**Results:**

In men, total cholesterol was inversely related to aPTT but positively associated with protein C activity. HDL cholesterol was inversely related to aPTT and fibrinogen. LDL cholesterol, non-HDL cholesterol, and triglycerides showed a positive association with protein C and protein S activity.

In women, LDL-cholesterol, total cholesterol, and non-HDL-cholesterol were positively related to AT III concentrations and protein C and S activity. Additionally, non-HDL-cholesterol was positively associated with factor VIII activity. HDL cholesterol was inversely related to fibrinogen. Triglycerides showed a positive relationship with protein C activity.

**Conclusions:**

There seem to be sex differences regarding various associations between blood lipid levels and hemostatic factors. Further studies are needed to address the possible impact of these associations on cardiovascular risk and the underlying mechanisms.

**Supplementary Information:**

The online version contains supplementary material available at 10.1186/s12944-022-01757-0.

## Background

Dyslipidemia is a major risk factor for cardiovascular disease (CVD) [1]. Among others, elevated low-density lipoprotein cholesterol (LDL-c), decreased high-density lipoprotein cholesterol (HDL-c), and increased triglyceride (TG) levels in the blood are associated with an increased risk of coronary atherosclerosis [2, 3]. While LDL-c is considered the most important lipoprotein-based risk factor, recent epidemiological studies suggest that non-HDL-c may be superior to LDL-c in determining coronary heart disease (CHD) risk [4, 5].

A number of previous studies have examined the role of the hemostatic system in the manifestation of CVD [6–8] and have shown that systemic coagulation activity contributes to acute coronary events [9]. For example, associations between fibrinogen [10], Faktor VIII [11], and protein C and protein S [12] and incident CHD were found. Certain studies evaluated components of the blood coagulation system in subjects with familial combined hyperlipidemia, who have a substantially increased risk of CVD, and reported increased coagulation activity in these individuals [13]. Finally, other epidemiological studies have demonstrated a link between various coagulation factors and lipid parameters [8, 14].

Previous studies postulated that a hypercoagulable state was present in individuals with hypercholesterolemia [15]. It has been shown that blood lipids are associated with prothrombotic and endothelial-damaging properties [16]. Circulating lipids affect the vascular endothelium, platelets, and coagulation factors. They are associated with an alteration in the expression and/or function of thrombotic, fibrinolytic and rheological factors [16].

Data from large studies including men and women from the general population examining possible associations between different blood lipid parameters and a variety of coagulation factors are still scarce. Therefore, in the present study, sex-specific associations between LDL-c, HDL-c, non-HDL c, total cholesterol, and TG levels and the parameters activated partial thromboplastin time (aPTT), fibrinogen, D-dimers, antithrombin III (AT III), protein C, protein S, and factor VIII in the adult general population were investigated.

## Methods

### Study sample

The KORA (Cooperative Health Research in the Region of Augsburg, Germany) platform was established in 1996 and succeeded and advanced the MONICA study (Monitoring of trends and determinants in cardiovascular disease). The KORA cohort study consists of four cross-sectional baseline surveys (S1 1984/85, S2 1989/90, S3 1994/95 and S4 1999/2001, S1-S3 under the MONICA label [17]); since then, all study participants have been followed-up. In early 2018, a follow-up study called “KORA-Fit” started and was conducted in mid-2019. All living attendees of the KORA cohort born between 1945 and 1964 who agreed to be recontacted were invited for a new examination (*n* = 3059 or 64.4% of all appropriate participants).

In the present analysis, a subgroup of all KORA-Fit study participants was considered, meaning participants who took part in the S4 baseline survey (*n* = 1394 eligible persons). Of those, 856 S4 participants (61.4%) also took part in the KORA-Fit examination. Overall, 805 participants (378 men, 427 women) with available data on hemostatic parameters could be included in the analysis.

The Ethics Committee of the Bavarian Chamber of Physicians gave its approval for the study (KORA-Fit EC No 17040). All study participants gave written informed consent, and the study was performed in accordance with the Declaration of Helsinki.

### Data collection

The study participants were interviewed by certified and trained study nurses on education/socioeconomic status, lifestyle factors, and medication use. In addition, a standardized medical examination was undertaken that included fasting blood sampling. Height and weight were measured, and body mass index (BMI) was calculated as weight in kilograms divided by height in m^2^. Blood pressure was measured with an automated oscillometric device (HEM-705CP, Omron Corporation, Tokyo, Japan) after a five-minute rest while sitting. Education years up to 10 years were categorized as “low“, over 10 years of schooling/studying as “high “education levels. A history of diabetes was categorized as yes or no. A participant was defined as physically active if he/she participated in sports in summer and winter and for more than 1 h per week in at least one season [18, 19]. Alcohol intake was calculated in grams per day [18]. Smoking status was classified into smoker, ex smoker and never smoker [18, 20]. Detailed information on the data collection, examination processes, and variable definitions in the KORA studies is described in detail elsewhere [18].

### Laboratory measurements

All hemostatic factors were measured in citrate plasma. During blood sampling, the patients were in an overnight fasting state. The samples were then processed and stored at − 80 °C until analysis. The parameter aPTT (reference value 26–36 sec) was determined photometrically (Pathromtin SL, Siemens Healthcare). AT III activity (ref. value 78–113%) was measured via a chromogenic activity assay (Innovance Antithrombin-Assay, Siemens Healthcare). Fibrinogen (ref. value 210–400 mg/dl) was quantified photometrically and turbidimetrically (Multifibren U, Siemens Healthcare). D-dimers (ref. value < 500 μg/L) were analyzed using a particle-enhanced immunoturbidimetric assay (Innovance D-Dimer Kit, Siemens Healthcare).

Protein C and S activity (ref. value for protein C 70–140%; ref. value for protein S 73–130% for men, 52–126% for women) were determined by photometry (Berichrom Protein C, Siemens Healthcare; Hemoclot Protein S). Factor VIII activity (ref. value 70–150%) was measured by photometry (coagulation factor VIII deficient plasma reagents used with Pathromtin SL reagents, Siemens Healthcare). All measurements, except for the protein S activity (CaoChrom analyzer (Wien, Austria)), were performed on a Siemens BCS-XP analyzer (Siemens, Eschborn, Germany).

After overnight fasting, serum blood samples were collected and kept at room temperature until centrifugation. Serum was separated after 30 minutes. Samples were assayed immediately at the laboratory of the University Hospital Großhadern (Ludwig-Maximilians Universität, München). Serum total cholesterol and HDL-c were measured enzymatically on a Cobas 8000 c702 Roche chemistry analyzer (Hoffmann-La Roche AG, Basel/Switzerland). The Friedewald formula was used for calculating LDL-c [21]. Non-HDL-c was calculated via subtraction of HDL-c from total cholesterol. Serum triglycerides were determined by an enzymatic color test (according to Trinder) (Hoffmann-La Roche AG Basel/Switzerland) on a Cobas 8000 c702 (Hoffmann-La Roche AG Basel/Switzerland).

### Statistical analysis

Continuous variables were described by means ± standard deviations (SD) in the case of normal distribution. Nonnormally distributed variables were given as median and interquartile range (IQR). The Shapiro–Wilk test was used to check if the data were normally distributed. Means of normally distributed variables were compared by the two-sided t test. Median values (not normally distributed variables) were compared by the Mann–Whitney U test. Categorial variables were compared via Fisher’s exact test.

Multivariable linear regression models were used to investigate possible associations between the different serum lipid parameters (continuous variables) and aPTT, AT III, fibrinogen, D-dimer, protein C, protein S, and factor VIII. Participants taking anticoagulative medication were excluded from the analyses. The models were adjusted for the following confounders: BMI, age, sex (only in the total sample), education years, alcohol consumption, systolic blood pressure, smoking status, history of diabetes, and intake of lipid-lowering drugs. We performed additional analyses to account for clinical perspective and interpretation, using the lipid parameters in a binary way (abnormal vs. normal).

It was investigated whether the exposure-outcome associations (that is, lipid-coagulation factor associations) were modified by sex or age using formal tests for interaction (significance level 5%). The linearity assumption was tested by adding additional second-degree polynomial transformations of continuous covariates. Multicollinearity and heteroscedasticity were assessed by calculating the variance inflation factor and performing the Breusch–Pagan test, respectively. Finally, the assumption of normally distributed residuals was ensured by visual assessment of the respective Q-Q plots. *P* values < 0.05 were considered statistically significant. The statistical software IBM SPSS, version 28, was used for data analysis.

## Results

Table 1 shows the sex-specific characteristics of the study sample. The mean age was the same for men and women (63.1 years). On average, men had a higher BMI (28.4 vs. 27.4) and were more often highly educated (68% vs. 60%) than women. Smoking status also differed between the sexes. While 65.6% of all men were smokers or ex-smokers, this was the case for only 50.8% of all women.

**Table 1:** Sex-specific characteristics, serum lipids, and hemostatic factors (means ± SDs or median and IQR): the population-based KORA-Fit Study

	Participants (***n*** = 805)	Units	Ref. values	MEN (***n*** = 378)	WOMEN (***n*** = 427)	***p*** value
Age	805 (0 missings)	years		63.1 (5.8)	63.1 (5.5)	0.942
BMI	805 (0 missings)	kg/m^2^		28.4 (4.2)	27.4 (5.2)	0.003
Systolic blood pressure	803 (2 missings)	mmHg		130.2 (15.3)	120.2 (16.5)	< 0.001
Education	805 (0 missings)	education ≤10 yrs		118 (31.2)	171 (40.0)	0.010
		education > 10 yrs		260 (68.8)	256 (60.0)	
Diabetes	803 (2 missings)	yes		36 (9.5)	33 (7.7)	0.381
		no		342 (90.5)	392 (91.8)	
Physical activity	805 (0 missings)	yes		261 (69.0)	304 (71.2)	0.537
		no		117 (31.0)	123 (28.8)	
Smoking status	805 (0 missings)	Smoker		56 (14.8)	54 (12.6)	< 0.001
		Ex smoker		192 (50.8)	163 (38.2)	
		Never smoker		130 (34.4)	210 (49.2)	
Alcohol intake	804 (1 missing)	g/day		5.7 (0.0; 21.5)	6.6 (0.0; 22.9)	0.147
aPTT	803 (2 missings)	sec	26–36	31.5 (3.4)	0.6 (3.2)	< 0.001
Antithrombin III activity	804 (1 missing)	%	83–118	99.4 (10.3)	104.9 (10.4)	< 0.001
Fibrinogen	758 (47 missings)	mg/dl	210–400	298.9 (62.8)	305.2 (60.2)	0.163
D-dimers	805 (0 missing)	ng/ml	≤500	408.0 (314.8; 563.0)	405.5 (306.0; 554.0)	0.642
Protein C activity	805 (0 missing)	%	70–140	119.9 (17.4)	127.9 (17.5)	< 0.001
Protein S activity	789 (16 missings)	%	m 73–130, w 52–126	137.1 (38.5)	120.9 (27.6)	< 0.001
Factor VIII activity	804 (1 missing)	%	70–150	122.6 (36.9)	124.9 (34.4)	0.371
Total cholesterol	805 (0 missing)	mg/dl	normal 0–200	190 (50.3)	132 (30.9)	< 0.001
			high > 200	188 (49.7)	295 (69.1)	
HDL cholesterol	805 (0 missing)	mg/dl	low ≤45	111 (29.4)	26 (6.1)	< 0.001
			normal > 45	267 (70.6)	401 (93.9)	
LDL cholesterol (Friedewald)	760 (45 missings)	mg/dl	normal ≤160	319 (86.7)	354 (84.1)	0.315
			high > 160	49 (13.3)	67 (15.9)	
Non-HDL cholesterol	805 (0 missing)	mg/dl	normal ≤130	145 (38.4)	137 (32.1)	0.065
			high > 130	233 (61.6)	290 (67.9)	
Triglycerides	805 (0 missing)	mg/dl	normal 0–200	325 (86.0)	394 (92.3)	0.004
			high > 200	53 (14.0)	33 (7.7)	

The aPTT values in men were higher than those in women (31.5 sec vs. 30.6 sec). Furthermore, protein C activity was higher in women (119.9% vs. 127.9%), while protein S activity was higher in men (137.1% vs. 120.9%). All other coagulation parameters showed no notable differences between males and females.

Total cholesterol, HDL cholesterol, LDL cholesterol, and triglycerides were different between the two sexes: total cholesterol levels in men were significantly lower than in women, as well as HDL-c and LDL-c. TGs were higher in men than in women.

In the whole population, LDL-c, total cholesterol, and non-HDL-c were positively associated with AT III, protein C and protein S activity. HDL-c was inversely related to aPTT and fibrinogen. Furthermore, there was a positive association between triglycerides and protein C and protein S activity (see Table 2).

**Table 2:** Results of the linear regression (ß value, 95% CI, *p* value) on the associations between serum lipids and coagulation parameters in both sexes (KORA-Fit)

	Total cholesterol mg/dl	HDL cholesterol mg/dl	LDL cholesterol (Fried) mg/dl	non-HDL cholesterol mg/dl	Triglycerides mg/dl
aPTT	−0.002 (− 0.009, 0.004)	−0.022 (− 0.037, − 0.006)	0.001 (− 0.006, 0.008)	0.002 (− 0.005, 0.008)	0.001 (− 0.003, 0.005)
	0.523	0.005	0.822	0.584	0.571
AT III	0.039 (0.019, 0.059)	0.023 (− 0.024, 0.070)	0.037 (0.015, 0.059)	0.034 (0.014, 0.053)	0.002 (−0.008, 0.011)
	< 0.001	0.334	< 0.001	< 0.001	0.753
Fibrinogen	−0.015 (− 0.137, 0.106)	− 0.505 (− 0.785, − 0.225)	0.084 (− 0.051, 0.219)	0.076 (− 0.044, 0.196)	0.001 (− 0.068, 0.071)
	0.804	< 0.001	0.220	0.212	0.975
D-dimers	0.515 (− 0.299, 1.329)	−1.083 (−2.979, 0.813)	0.625 (− 0.242, 1.491)	0.698 (− 0.107, 1.501)	0.151 (− 0.244, 0.546)
	0.215	0.263	0.157	0.089	0.453
Protein C	0.156 (0.124, 0.189)	0.064 (−0.016, 0.144)	0.139 (0.102, 0.175)	0.141 (0.108, 0.173)	0.033 (0.016, 0.049)
	< 0.001	0.118	< 0.001	< 0.001	< 0.001
Protein S	0.150 (0.084, 0.215)	0.012 (−0.141, 0.165)	0.125 (0.053, 0.196)	0.143 (0.078, 0.208)	0.061 (0.029, 0.092)
	< 0.001	0.876	< 0.001	< 0.001	< 0.001
Factor VIII	0.069 (0.001, 0.137)	0.068 (−0.091, 0.227)	0.055 (−0.020, 0.131)	0.055 (− 0.012, 0.123)	0.019 (− 0.014, 0.052)
	0.046	0.400	0.150	0.106	0.260

Participants with anticoagulative medication were excluded from the linear regression analyses

Independent variables: lipid parameters (continuously). Adjusted for age, sex, BMI, education (≤/> 10 years), diabetes, smoking (yes/no/never), systolic blood pressure, and intake of antihypertensive medication

*aPTT* activated partial prothrombin time

Due to significant interactions with sex indicating different effect sizes for men and women in the multivariable linear regression models, the analyses were stratified by sex. In men, total cholesterol was inversely associated with aPTT and positively associated with protein C activity. HDL-c was inversely related to both aPTT and fibrinogen. LDL-c, non-HDL-c and TG values showed positive associations with protein C and protein S activity (see Table 3).

**Table 3:** Results of the linear regression (ß value, 95% CI, *p* value) on the associations between serum lipids and coagulation parameters in men (KORA-Fit)

	Total cholesterol mg/dl	HDL cholesterol mg/dl	LDL cholesterol mg/dl	Non-HDL cholesterol mg/dl	Triglycerides mg/dl
aPTT	− 0.011 (− 0.022, − 0.001)	−0.039 (− 0.065, − 0.013	−0.008 (− 0.020, 0.003)	−0.005 (− 0.016, 0.005)	0.000 (− 0.004, 0.005)
	0.030	0.004	0.164	0.304	0.963
AT III	0.005 (−0.025, 0.035)	−0.030 (− 0.107, 0.047)	0.012 (− 0.020, 0.045)	0.009 (− 0.020, 0.038)	−0.002 (− 0.013, 0.010)
	0.748	0.446	0.450	0.547	0.769
Fibrinogen	−0.127 (− 0.318, 0.063)	−0.696 (−1.194, − 0.198)	−0.037 (− 0.248, 0.173)	−0.026 (− 0.213, 0.161)	0.012 (− 0.081, 0.105)
	0.189	0.006	0.728	0.784	0.799
D-dimers	0.621 (−0.785, 2.027)	−2.894 (−6.491, 0.702)	0.507 (− 0.940, 1.955)	1.008 (− 0.360, 2.376)	0.286 (− 0.241, 0.813)
	0.386	0.114	0.491	0.148	0.286
Protein C	0.125 (0.074, 0.176)	0.015 (−0.121, 0.150)	0.112 (0.056, 0.169)	0.116 (0.066, 0.166)	0.023 (0.004, 0.043)
	< 0.001	0.828	< 0.001	< 0.001	0.020
Protein S	0.238 (0.121, 0.355)	0.059 (−0.241, 0.360)	0.173 (0.046, 0.300)	0.216 (0.102, 0.330)	0.060 (0.017, 0.103)
	0.121	0.698	0.008	< 0.001	0.006
Factor VIII	0.043 (−0.065, 0.152)	0.248 (−0.030, 0.526)	0.001 (− 0.119, 0.121)	0.005 (− 0.101, 0.112)	0.011 (− 0.030, 0.052)
	0.434	0.081	0.984	0.920	0.593

Participants with anticoagulative medication were excluded from the linear regression analyses

Independent variables: lipid parameters (continuously). Adjusted for age, BMI, education (≤/> 10 years), diabetes, smoking (yes/no/never), systolic blood pressure, and intake of antihypertensive medication

*aPTT* activated partial prothrombin time

In women, total, LDL and non-HDL cholesterol were positively related to AT III concentrations as well as protein C and protein S activity. In addition, non-HDL-c was positively associated with factor VIII activity. For men, HDL-c was inversely related to fibrinogen. TG concentrations showed a positive association with protein C activity (see Table 4).

**Table 4:** Results of the linear regression (ß value, 95% CI, *p* value) on the associations between serum lipids and coagulation parameters in women (KORA-Fit)

	Total cholesterol mg/dl	HDL cholesterol mg/dl	LDL cholesterol mg/dl	Non-HDL cholesterol mg/dl	Triglycerides mg/dl
aPTT	0.004 (−0.004, 0.013)	−0.013 (− 0.032, 0.005)	0.007 (− 0.002, 0.017)	0.007 (− 0.001, 0.016)	0.003 (− 0.003, 0.009)
	0.313	0.155	0.129	0.094	0.294
AT III	0.065 (0.038, 0.092)	0.053 (−0.007, 0.113)	0.056 (0.026, 0.087)	0.054 (0.026, 0.081)	0.009 (−0.010, 0.028)
	< 0.001	0.081	< 0.001	< 0.001	0.360
Fibrinogen	0.050 (−0.110, 0.211)	−0.401 (− 0.739, − 0.063)	0.164 (− 0.016, 0.345)	0.141 (− 0.020, 0.301)	−0.027 (− 0.136, 0.083)
	0.538	0.020	0.074	0.086	0.631
D-dimers	0.474 (−0.482, 1.430)	0.214 (−1.845, 2.272)	0.621 (−0.446, 1.687)	0.428 (− 0.528, 1.385)	−0.164 (− 0.830, 0.502)
	0.330	0.838	0.253	0.379	0.629
Protein C	0.180 (0.137, 0.223)	0.093 (−0.007, 0.193)	0.160 (0.110, 0.209)	0.160 (0.116, 0.204)	0.059 (0.027, 0.091
	< 0.001	0.069	< 0.001	< 0.001	< 0.001
Protein S	0.083 (0.009, 0.157)	0.009 (−0.149, 0.167)	0.092 (0.011, 0.173)	0.081 (0.008, 0.155)	0.041 (−0.010, 0.092)
	0.027	0.912	0.026	0.031	0.111
Factor VIII	0.088 (−0.001, 0.178)	−0.032 (− 0.225, 0.162)	0.095 (− 0.005, 0.196)	0.095 (0.006, 0.185)	0.042 (− 0.021, 0.104)
	0.053	0.749	0.061	0.037	0.192

Participants with anticoagulative medication were excluded from the linear regression analyses

Independent variables: lipid parameters (continuously). Adjusted for age, BMI, education (≤/> 10 years), diabetes, smoking (yes/no/never), systolic blood pressure, and intake of antihypertensive medication

*aPTT* activated partial prothrombin time

All findings were supported by regression models using the lipid parameters in a binary way (abnormal versus normal) (see Additional file 1, Tables 1, 2 and 3).

## Discussion

The present study showed that commonly measured hemostatic factors were associated with several blood lipid markers in individuals from the general population. There were significant sex-specific differences; in particular, more notable associations, especially with non-HDL-c, could be observed in women. However, commonalities between the sexes could be found in the inverse relationship of HDL-c and fibrinogen and positive relationships of LDL-c as well as non-HDL-c with protein C.

Protein C and its cofactor protein S are vitamin K-dependent coagulation inhibitors and play an important role in fibrinolytic processes [22]. In both sexes, these two coagulation factors showed similar associations with total cholesterol, LDL-c, and non-HDL-c; TGs were associated with both coagulation inhibitors in men, but in women, they were only related to protein C. A positive correlation of protein C with LDL-c was also found in the ARIC Study [23], and in a Polish study, protein C activity was associated with hypercholesterolemia [24]. Women had higher protein C activity than men, and protein S activity was significantly higher in men. Protein C activity is known to change in women taking oral contraceptives or who are pregnant [25]. Since this was not the case in the present study, because the mean age of the women was 63.1 years, it could be assumed that in postmenopausal women, protein C activity might be higher compared to men. Similar to protein C and protein S, AT III also has antithrombotic properties and inhibits serine protease factors II, IX, X, XI, and XII [26]. In the present study, the AT III activity in women was significantly higher than that in men. Furthermore, in females, a significant association between total cholesterol, LDL-c and non-HDL-c and AT III was observed, but none of the lipid parameters were associated with AT III in men. This result is in accordance with findings from the ARIC study [27], which observed higher AT III concentrations in women than in men. Furthermore, in a study from Japan including hyperlipidemic and normolipidemic elderly individuals, in both groups, serum total cholesterol correlated positively with AT III activity [28]. In contrast, there was no notable association between LDL-c and AT III in the ARIC study [27]. A Polish study including a random sample of community-dwelling older individuals also did not find an association between AT III activity and total cholesterol and LDL-c [29]. In addition, total cholesterol was not associated with AT III in a study on 130 healthy adults aged 20–60 years from Poland [24].

Contrary to prior studies [27, 30, 31], fibrinogen values did not differ between men and women in the present investigation. A number of previous studies [30–33], but not all [34, 35], reported a positive association between fibrinogen and LDL-c. In the present study, no independent association between fibrinogen and LDL-c was observed, but an inverse relationship between fibrinogen and HDL-c in both sexes was found. This result agrees with findings from ARIC [8, 14] but could not be confirmed by other investigations [31, 32, 36]. D-dimer levels did not differ between males and females and showed no significant association with any of the different lipid parameters. In contrast, in the Dallas Heart Study, in linear regression analyses, D-dimer, a marker of fibrin formation and lysis, was significantly higher in females than in males even after multivariable adjustment for a number of covariables, including body composition [37].

In the present study, a significant relationship between factor VIII activity and non-HDL-c was observed in women. No other lipid parameters showed a relationship with factor VIII. Furthermore, no notable differences in factor VIII concentrations were found between the two sexes. This is in contrast to the ARIC study, where the mean levels of Factor VIII were higher in men than in women [23]. In addition, in ARIC in univariable analysis, factor VIII was negatively associated with HDL-c and positively associated with LDL-c [23]. In the third Glasgow MONICA Survey II [14], factor VIII correlated with cholesterol in women but not men. There are no prior studies on the association between non-HDL-c and hemostatic factors. Thus, the findings of the present study have to be confirmed by further investigations.

An inverse association between HDL-c and aPTT, a summary index for intrinsic and common pathways, was found in men but not in women in the present study. Furthermore, in males but not females, total cholesterol was inversely related to aPTT, which is a widely used routine screening test of the coagulation system. It could be shown that patients with hypercholesterolemia had shorter aPTT values than patients with lower cholesterol levels [38]. The results of the present study in men are consistent with this finding, but it is unclear why women do not show this relationship. Furthermore, contrary to this finding, no correlation between HDL-c and aPTT was observed in a prior study [39]. In contrast to the study of Chan et al. [40], where TGs were also related to aPTT, in the present study, such an association could not be observed.

As an underlying mechanism for the association between blood lipid levels and hemostasis factors, a previous study suggested that hypercholesterolemia may affect the regulation of blood coagulation via tissue factor pathway inhibitor (TFPI) [41]. TFPI, formerly also called lipoprotein-associated coagulation inhibitor [42] or extrinsic pathway inhibitor [43], is a protease inhibitor of the first steps of the extrinsic blood coagulation pathway. Increased TFPI activity was found in patients with hyperlipidemia [44], and there are positive correlations between plasma TFPI activity and total cholesterol [45] as well as LDL cholesterol levels [44]. In addition, studies have shown that hyperlipidemia may be associated with decreased activation of protein C, which in its activated form can inhibit several steps in the blood coagulation pathways.

### Comparisons with other studies and what does the current work add to the existing knowledge

Prior studies observed possible effects of dyslipidemia on the coagulation system [23, 24, 27, 28, 30–35]. The present study found that sex is a moderator responsible for different effects in men and women regarding the relationships between lipid concentrations and coagulation factors.

### Study strengths and limitations

The strengths of this study are the sample size, the availability of standardized measured laboratory data, information on medication intake, and standardized assessed cardiovascular risk factors. There are also certain limitations. The analysis was based on a follow-up study of a population-based study. Thus, it could be assumed that the participants are not representative of the initial population-based sample. Selection bias that may have affected the present results cannot be entirely excluded. This study included German subjects born between 1945 and 1964, so the results do not apply to other age groups and individuals of other ethnic origins.

## Conclusions

There appear to be sex-specific differences regarding various associations between blood lipid levels and hemostatic factors in individuals from the general population. Therefore, in clinical practice, the role of lipid concentrations in the pathogenesis of venous thromboembolism (VTE) should not be neglected. Patients at high risk for developing venous thromboembolism could benefit from a reduction in lipid concentrations, e.g., by treatment with lipid-lowering drugs.

## Supplementary Information

**Additional file 1: Table 1.** Results of the linear regression (ß value, 95% CI, *p* value) on the associations between categorized serum lipids (abnormal vs. normal) and coagulation parameters in both sexes (KORA-Fit). **Table 2.** Results of the linear regression (ß value, 95% CI, p value) on the associations between categorized serum lipids (abnormal vs. normal) and coagulation parameters in men (KORA-Fit). **Table 3.** Results of the linear regression (ß value, 95% CI, p value) on the associations between categorized serum lipids (abnormal vs. normal) and coagulation parameters in women (KORA-Fit).

## Data Availability

The data that support the findings of this study are available from Helmholtz Zentrum München, but restrictions apply to the availability of these data, which are not publicly available. Data are, however, available from the authors upon reasonable request and with permission of Helmholtz Zentrum München.

## Abbreviations

aPTT: Activated partial thromboplastin time
AT III: Antithrombin III
BMI: Body mass index
CHD: Coronary heart disease
CVD: Cardiovascular disease
HDL-c: High-density lipoprotein cholesterol
IQR: Interquartile range
KORA: Cooperative Health Research in the Region of Augsburg, Germany
LDL-c: Low-density lipoprotein cholesterol
Non-HDL-c: Non-HDL cholesterol
SD: Standard deviation
TG: Triglycerides
VTE: Venous thromboembolism
