# Supplementary material for: Sex-specific associations between serum lipids and hemostatic factors: the cross-sectional population-based KORA-fit study
Source: Lipids Health Dis. 2022 Dec 21;21:143. doi: 10.1186/s12944-022-01757-0 (PMC9768912; doi:10.1186/s12944-022-01757-0)
Supplement: Supplementary file 1 — Additional file 1: Table 1. Results of the linear regression (ß value, 95% CI, p value) on the associations between categorized serum lipids (abnormal vs. normal) and coagulation parameters in both sexes (KORA-Fit). Table 2. Results of the linear regression (ß value, 95% CI, p value) on the associations between categorized serum lipids (abnormal vs. normal) and coagulation parameters in men (KORA-Fit). Table 3. Results of the linear regression (ß value, 95% CI, p value) on the associations between categorized serum lipids (abnormal vs. normal) and coagulation parameters in women (KORA-Fit). [file 12944_2022_1757_MOESM1_ESM.docx]

**Additional file 1**

**Sex-specific associations between serum lipids and hemostatic factors: the cross-sectional population-based KORA-Fit Study**

Johannes Vogel von Falckenstein, Dennis Freuer, Annette Peters, Margit Heier, Daniel Teupser, Jakob Linseisen, Christa Meisinger

**Table 1:** Results of the linear regression (ß value, 95% CI, p value) on the associations between categorized serum lipids (abnormal vs. normal) and coagulation parameters in both sexes (KORA-Fit)

|  | | Total cholesterol mg/dl | HDL cholesterol mg/dl | LDL cholesterol mg/dl | Non-HDL cholesterol mg/dl | | Triglycerides mg/dl |
| --- | --- | --- | --- | --- | --- | --- | --- |
| aPTT | | 0.079 (-0.414, 0.571) | -1.017 (-1.711, -0.323) | 0.075 (-0.584, 0.734) | 0.271 (-0.220, 0.763) | | 0.451 (-0.342, 1.245) |
|  | | 0.754 | 0.004 | 0.824 | 0.279 | | 0.265 |
| AT III | | 2.140 (0.622, 3.658) | 1.222 (-0.931, 3.375) | 2.024 (0.006, 4.041) | 1.186 (-0.338, 2.710) | | -0.378 (-2.817, 2.060) |
|  | | 0.006 | 0.266 | 0.049 | 0.127 | | 0.761 |
| Fibrinogen | | -4.988 (-14.142, 4.165) | -11.521 (-24.464, 1.421) | 12.390 (0.233, 24.547) | -1.638 (-10.812, 7.535) | | -7.459 (-22.255, 7.337) |
|  | | 0.285 | 0.081 | 0.046 | 0.726 | | 0.323 |
| D-dimers | | 51.421 (-10.210, 113.052) | -77.451 (-164.444, 9.542) | 40.168 (-39.169, 119.506) | 62.416 (0.826, 124.007) | | 28.101 (-70.577, 126.780) |
|  | | 0.102 | 0.081 | 0.321 | 0.047 | | 0.576 |
| Protein C | | 8.427 (5.890, 10.964) | 1.730 (-1.948, 5.409) | 8.142 (4.713, 11.571) | 7.179 (4.622, 9.736) | | 5.824 (1.677, 9.971) |
|  | | <0.001 | 0.356 | <0.001 | <0.001 | | 0.006 |
| Protein S | | 9.979 (5.035, 14.923) | 1.244 (-5.820, 8.309) | 5.818 (-0.770, 12.407) | 10.000 (5.052, 14.948) | | 10.223 (2.292, 18.154) |
|  | | <0.001 | 0.730 | 0.083 | <0.001 | | 0.012 |
| Factor VIII | | 1.172 (-4.014, 6.357) | 4.338 (-2.975, 11.650) | 4.143 (-2.794, 11.080) | -0.148 (-5.335, 5.040) | | 4.310 (-3.971, 12.591) |
|  | | 0.657 | 0.245 | 0.241 | 0.955 | | 0.307 |
| aPTT = activated partial prothrombin time  Participants with anticoagulative medication were excluded from the linear regression analyses. | | | | |  |  |  |
| Independent variables: lipid parameters (abnormal vs. normal). Adjusted for age, BMI, sex, education (≤/> 10 years), diabetes, smoking (yes/no/never), systolic blood pressure, and intake of antihypertensive medication.  Total cholesterol: normal 0-200 mg/dl, high >200 mg/dl; HDL cholesterol: low ≤45 mg/dl, normal >45 mg/dl; LDL cholesterol normal ≤160 mg/dl, high >160 mg/dl; non-HDL cholesterol: normal ≤130 mg/dl, high >130 mg/dl; Triglycerides normal 0-200 mg/dl, high >200 mg/dl. | | | | |  |  |  |

**Table 2:** Results of the linear regression (ß value, 95% CI, p value) on the associations between categorized serum lipids (abnormal vs. normal) and coagulation parameters in men (KORA-Fit)

|  | | Total cholesterol mg/dl | HDL cholesterol mg/dl | LDL cholesterol mg/dl | Non-HDL cholesterol mg/dl | | Triglycerides mg/dl |
| --- | --- | --- | --- | --- | --- | --- | --- |
| aPTT | | -0.313 (-1.045, 0.420) | -0.843 (-1.695, 0.009) | -0.264 (-1.333, 0.804) | -0.098 (-0.848, 0.651) | | 0.405 (-0.694, 1.504) |
|  | | 0.401 | 0.052 | 0.627 | 0.796 | | 0.469 |
| AT III | | 0.019 (-2.134, 2.173) | -1.153 (-3.660, 1.354) | 0.096 (-3.003, 3.195) | -1.395 (-3.598, 0.809) | | -1.388 (-4.564, 1.787) |
|  | | 0.986 | 0.366 | 0.951 | 0.214 | | 0.390 |
| Fibrinogen | | -15.562 (-29.067, -2.056) | -24.630 (-40.271, -8.988) | 15.609 (-3.805, 35.023) | -12.536 (-26.475, 1.403) | | -5.060 (-25.754, 15.634) |
|  | | 0.024 | 0.002 | 0.115 | 0.078 | | 0.631 |
| D-dimers | | 28.853 (-70.849, 128.554) | -122.675 (-238.177, -7.172) | -28.170 (-163.555, 107.216) | 79.928 (-21.979, 181.836) | | 50.398 (-96.726, 197.522) |
|  | | 0.570 | 0.037 | 0.683 | 0.124 | | 0.501 |
| Protein C | | 8.166 (4.535, 11.797) | -1.374 (-5.722, 2.973) | 6.074 (0.696, 11.452) | 7.128 (3.376, 10.879) | | 4.521 (-0.968, 10.010) |
|  | | <0.001 | 0.534 | 0.027 | <0.001 | | 0.106 |
| Protein S | | 12.704 (4.486, 20.925) | -0.471 (-10.234, 9.291) | 11.994 (0.033, 23.956) | 11.812 (3.354, 20.269) | | 12.262 (0.014, 24.511) |
|  | | 0.003 | 0.924 | 0.049 | 0.006 | | 0.050 |
| Factor VIII | | -0.684 (-8.450, 7.083) | 2.627 (-6.411, 11.665) | 2.906 (-8.386, 14.199) | -1.583 (-9.546, 6.381) | | -1.241 (-12.693, 10.212) |
|  | | 0.863 | 0.568 | 0.613 | 0.696 | | 0.831 |
| aPTT = activated partial prothrombin time  Participants with anticoagulative medication were excluded from the linear regression analyses. | | | | |  |  |  |
| Independent variables: lipid parameters (abnormal vs. normal). Adjusted for age, BMI, education (≤/> 10 years), diabetes, smoking (yes/no/never), systolic blood pressure, and intake of antihypertensive medication.  Total cholesterol: normal 0-200 mg/dl, high >200 mg/dl; HDL cholesterol: low ≤45 mg/dl, normal >45 mg/dl; LDL cholesterol normal ≤160 mg/dl, high >160 mg/dl; non-HDL cholesterol: normal ≤130 mg/dl, high >130 mg/dl; Triglycerides normal 0-200 mg/dl, high >200 mg/dl. | | | | |  |  |  |

**Table 3:** Results of the linear regression (ß value, 95% CI, p value) on the associations between categorized serum lipids (abnormal vs. normal) and coagulation parameters in women (KORA-Fit)

|  | | Total cholesterol mg/dl | HDL cholesterol mg/dl | LDL cholesterol mg/dl | Non-HDL cholesterol mg/dl | | Triglycerides mg/dl |
| --- | --- | --- | --- | --- | --- | --- | --- |
| aPTT | | 0.392 (-0.285, 1.069) | 1.784 (-3.197, -0.370) | 0.178 (-0.671, 1.026) | 0.540 (-00.118, 1.198) | | 0.498 (.0,705, 1.700) |
|  | | 0.256 | 0.014 | 0.681 | 0.108 | | 0.416 |
| AT III | | 4.092 (1.924, 6.259) | 8.090 (3.524, 12.655) | 3.281 (0.556, 6.005) | 3.443 (1.320, 5.566) | | 1.193 (-2.725, 5.111) |
|  | | <0.001 | <0.001 | 0.018 | 0.002 | | 0.550 |
| Fibrinogen | | 4.700 (-7.927, 17.328) | 19.080 (-7.197, 45.357) | 9.288 (-6.565, 25.140) | 8.679 (-3.597, 20.955) | | -8.851 (-31.023, 13.322) |
|  | | 0.465 | 0.154 | 0.250 | 0.165 | | 0.433 |
| D-dimers | | 72.329 (-3.166, 147.825) | -4.907 (-164.207, 154.394) | 80.593 (-14.128, 175.313) | 49.271 (-24.435, 122.977) | | 21.795 (-112.777, 156.368) |
|  | | 0.060 | 0.952 | 0.095 | 0.190 | | 0.750 |
| Protein C | | 8.709 (5.092, 12.236) | 9.478 (1.728, 17.228) | 9.776 (5.230, 14.322) | 7.197 (3.647, 10.748) | | 7.338 (0.783, 13.893) |
|  | | <0.001 | 0.017 | <0.001 | <0.001 | | 0.028 |
| Protein S | | 7.950 (2.100, 13.801) | 5.346 (-6.966, 17.658) | 2.515 (-4.814, 9.844) | 8.763 (3.070, 14.455) | | 7.191 (-3.218, 17.601) |
|  | | 0.008 | 0.394 | 0.500 | 0.003 | | 0.175 |
| Factor VIII | | 2.675 (-4.449, 9.798) | 7.776 (-7.181, 22.734) | 4.579 (-4.366, 13.523) | 1.052 (-5.891, 7.995) | | 13.318 (0.731, 25.904) |
|  | | 0.461 | 0.307 | 0.315 | 0.766 | | 0.038 |
| aPTT = activated partial prothrombin time  Participants with anticoagulative medication were excluded from the linear regression analyses. | | | | |  |  |  |
| Independent variables: lipid parameters (abnormal vs. normal). Adjusted for age, BMI, education (≤/> 10 years), diabetes, smoking (yes/no/never), systolic blood pressure, and intake of antihypertensive medication.  Total cholesterol: normal 0-200 mg/dl, high >200 mg/dl; HDL cholesterol: low ≤45 mg/dl, normal >45 mg/dl; LDL cholesterol normal ≤160 mg/dl, high >160 mg/dl; non-HDL cholesterol: normal ≤130 mg/dl, high >130 mg/dl; Triglycerides normal 0-200 mg/dl, high >200 mg/dl. | | | | |  |  |  |
